# Motor-based bodily self is selectively impaired in eating disorders

**DOI:** 10.1371/journal.pone.0187342

**Published:** 2017-11-01

**Authors:** Giovanna Cristina Campione, Gianluigi Mansi, Alessandra Fumagalli, Beatrice Fumagalli, Simona Sottocornola, Massimo Molteni, Nadia Micali

**Affiliations:** 1 Child Psychopathology Unit, Scientific Institute, IRCCS Eugenio Medea, Bosisio Parini, Italy; 2 Eating and Weight Disorders Program, Department of Psychiatry, Icahn School of Medicine at Mount Sinai, New York, United States of America; 3 Institute of Child Health, University College London, London, United Kingdom; Universita degli Studi di Udine, ITALY

## Abstract

**Background:**

Body representation disturbances in body schema (i.e. unconscious sensorimotor body representations for action) have been frequently reported in eating disorders. Recently, it has been proposed that body schema relies on adequate functioning of the motor system, which is strongly implicated in discriminating between one’s own and someone else’s body. The present study aimed to investigate the motor-based bodily self in eating disorders and controls, in order to examine the role of the motor system in body representation disturbances at the body schema level.

**Method:**

Female outpatients diagnosed with eating disorders (N = 15), and healthy controls (N = 18) underwent a hand laterality task, in which their own (self-stimuli) and someone else’s hands (other-stimuli) were displayed at different orientations. Participants had to mentally rotate their own hand in order to provide a laterality judgement. Group differences in motor-based bodily self-recognition—i.e. whether a general advantage occurred when implicitly processing self- vs. other-stimuli − were evaluated, by analyzing response times and accuracy by means of mixed ANOVAs.

**Results:**

Patients with eating disorders did not show a temporal advantage when mentally rotating self-stimuli compared to other-stimuli, as opposed to controls (F(1, 31) = 5.6, p = 0.02; eating disorders-other = 1092 ±256 msec, eating disorders-self = 1097±254 msec; healthy controls-other = 1239±233 msec, healthy controls -self = 1192±232 msec).

**Conclusion:**

This study provides initial indication that high-level motor functions might be compromised as part of body schema disturbances in eating disorders. Further larger investigations are required to test motor system abnormalities in the context of body schema disturbance in eating disorders.

## Introduction

Body representation disturbances are one of the central core symptoms of Eating Disorders (EDs) and may involve atypical processes at level of both the conscious perceptual, semantic—affective representations of the body (i.e. body image) and the functional, sensorimotor representations of body and space for action (i.e. body schema; [1–5]). Within the broader category of EDs, Anorexia Nervosa (AN) and Bulimia Nervosa (BN) are the two major conditions in which difficulties in body representation are described, and distress and concern about the body occur [6]. Despite the recognized impact of atypical body representation in EDs [7–10], however, neuro-functional underpinnings of such difficulties have not been fully elucidated. Furthermore, while several studies have extensively investigated body image disturbance in AN and BN (see for instance [11–14]), only a few studies specifically studied body schema and its functions.

According to the view that body schema is a dynamic representation interacting with the motor system for the control of action [15–19] − and underlies motor simulation as well [20–25] − Guardia and colleagues [26, 27] evaluated it in AN using a body-scale-action task, where participants were requested to image whether or not an aperture was wide enough for them to pass through. Patients with AN had a disturbance in anticipation of body-scaled action due to an overestimated body schema. Two further studies [28, 29] confirmed that patients with AN had the same difficulties when the action to pass through an aperture was actually executed, due to an enlarged body representation at the level of body schema resistant to the corrective feedback of action execution. Taken together, these data suggest a substantial impairment in body schema in patients with AN.

Laterality judgement tasks − where individuals have to simulate their own movements in order to provide a response [17, 20–22, 24, 25] − have also been used to assess body schema. Urgesi and colleagues [30] asked participants with BN to provide a laterality judgement of schematic front- and back-facing human body figures, and showed these patients had a reduced ability to manipulate body schema compared to controls. More specifically, patients with BN were impaired in providing laterality judgments on the front-facing stimuli when asked to simulate a motor rotation of their own body (i.e. motor mental rotation) in order to assume the perspective of the displayed body figure. This suggests a maladaptive representation of the bodily self.

Recent hypotheses in the field of motor cognition propose that body schema and bodily self are essentially motor in nature [15, 31, 32], and body schema does not only interact with the motor system as a representation merely derived from multiple sensorimotor inputs for control of action, but, on the contrary, it has its own motor intentional features [15, 19, 33, 34]. According to this view, body schema provides a repertoire of high-level motor functions (i.e. complex motor processes directly involved in cognition [4, 15, 34, 35]), like for instance the specification of motor potentialities in space, on which the bodily self (i.e. a “*pre-reflective consciousness of the body as one’s own body*”) is grounded [15]. According to this view Ferri and colleagues reported that motor simulation is crucial in order to recognize one’s own body [32]. Using a Hand Laterality Task (HLT; [24, 25]), where individuals are shown with hands displayed at different orientations, and are required to judge whether the hand shown is right or left to elicit a motor mental rotation, Ferri covertly presented a group of healthy participants with both their own and someone else’s hands. A Self-Advantage Effect (SAE; [36, 37]) emerged, i.e. an overall advantage in motor mental rotation of one’s own hand, as opposed to someone else’s hand. It has been well-established that motor mental rotation is based on motor simulation (i.e. mentally moving one’s own body part; [24, 25, 38–40]), as participants have to mentally simulate the rotation of their own hand to provide the response [17, 24]. Indeed, when a motor mental rotation occurs, Response Times (RTs) and accuracy respectively increase and decrease as a function of the increase in the amount of angular displacement between the observer’s real hand and the presented hand [24, 25, 38–40]. Ferri showed that SAE only arose when a motor mental rotation actually occurred (and an explicit recognition of one’s own hand was not required), but not when visual identity discrimination (and explicit bodily-self recognition) was directly required. Therefore, implicit bodily self-recognition crucially arises when we mentally simulate our own body movements, and motor simulation depends on body schema [20–25].

Based on this evidence, and with the aim to examine whether high-level motor functions may be compromised as part of body schema disturbances in EDs compared to healthy controls, in the present study we employed Ferri and colleagues’ [32] paradigm to add preliminary evidence to previous literature showing a body schema impairment in EDs by means of motor tasks [26–29]. While previous studies were in fact more specifically focused on studying more basic interactions between body schema and motor system for control of actions, in the present study we were interested in a more detailed understanding of the high-level motor nature of body schema disturbances in EDs. For this purpose, we carried out both a HLT with an implicit bodily self-recognition, and an identity discrimination visual task (IDVT), in which participants saw the same stimuli of the HLT, but were explicitly asked to visually recognize their own hands across a group of patients with EDs and a group of healthy controls. The IDVT provided therefore a control measurement to unambiguously ascribe putative group differences in the SAE of HLT to impairment in body schema-related functions. As the IDVT is a visual task, neither a motor mental rotation (with related modulations in RTs and accuracy) nor a SAE should be observed, in neither EDs nor the control group. On the other hand, we can expect a difference between patients with EDs and controls in the SAE of the HLT, if high-level motor functions are compromised as part of body schema disturbances in eating disorders.

## Material and methods

### Participants

Female outpatients with a diagnosis of EDs (N = 15) participated in the study. They were recruited from the Service for Eating Disorders of the Eugenio Medea Research Hospital, Bosisio Parini, Italy. Female Healthy Controls (HCs; N = 18) were recruited in collaboration with some local schools. Groups were matched on age, education, Body Mass Index (BMI), and handedness ([41], Table 1). Participants’ ages ranged from 15 to 21 years. Both left- and right-handed participants were included, as handedness does not affect the ability to perform a mental rotation when viewing a simple hand picture [25, 38]. Participants were excluded if they had distinctive features on the dorsal part of the hands, brain damage or any other neurological condition, and major general medical conditions likely to affect brain structure/function.

**Table 1:** Socio-demographic data, handedness, eating disorder- and body representation-related measures.

	HC group	EDs group	HC vs. EDs
	Mean (SD)	Mean (SD)	
**Age (years)**	17.3(1.9)	18.0(2.1)	t_(31)_ = 0.9, p = 0.4
**Education (years)**	11.6(1.7)	11.2(1.4)	t_(31)_ = 0.7, p = 0.5
**BMI**^a^**(kg/m**^**2**^**)**	20.5(2.1)	18.9(2.9)	t_(31)_ = 1.8, p = 0.08
**Illness duration (years)**	-	1.9(1.2)	-
**Handedness**	-	-	χ^2^_(1)_ = 0.7, p = 0.4; HC: R = 15; L = 3 EDs: R = 14; L = 1
**EDRC**^b^	40.1(26.1)	85.7(8.7)	**t**_**(27)**_ **=** **-6, p<0.001**
**BMI-ST**^c^	5.6(1.5)	8.6(3.3)	**t**_**(31)**_ **=** **-3.3, p<0.01**
**BAT-TOT**^d^	26.0(12.8)	66.4(13.6)	**t**_**(31)**_ **=** **-8.7 p<0.001**

Socio-demographic characteristics, handedness and Eating Disorder- and body representation-related measures in Healthy Control (HCs) and outpatients with Eating Disorders (EDs) are reported. Bold values index significant effects.

^a^BMI = Body Mass Index;

^b^EDRC = Eating Disorder Risk Scores (of Eating Disorder Inventory-3);

^c^BMI-ST = BMI-based Silhouettes Test scores;

^d^BAT-TOT = Body Attitude Test total scores.

ED diagnoses were established according to DSM-5 clinical criteria, and confirmed using the research version of the Structured Clinical Interview for DSM-IV axis I Disorders (SCID-I; [42]). Patients were included if they met criteria for AN (N_AN_ = 11; BMI range: 13–19.5 kg/m^2^) or BN (N_BN_ = 4; BMI range: 20–24 kg/m^2^), given that AN and BN share difficulties in body representation [6] and body schema [26–30]. Eight patients received the diagnosis of Restrictive Type AN, 3 the diagnosis of Binge-Eating/Purging Type AN. Two patients with AN were underweight, while all remaining patients with AN were weight-recovered. One patient with AN and one patient with BN had comorbidity with anxiety and mood disorders and were receiving pharmacological treatment. All patients were receiving individual psychotherapy at the time of data collection. Mean illness duration was 1.9 ± 1.2 years.

HCs were included if they had a BMI between 18 and 25 kg/m^2^, they were excluded if they were on a diet to lose weight at the time of data collection, had a psychiatric diagnosis, were on pharmacological medications, and had personal (or family) history of disordered eating behavior/ED, as assessed by the non-patient version of the SCID-I.

### Procedure

The Ethics Committee of Scientific Institute Eugenio Medea approved the study, which was conducted according to the declaration of Helsinki. All participants provided written informed consent prior to data collection (we obtained parental written informed consent for minors who participated in the study).

The HLT and an IDVT were administered by a trained neuropsychologist, and RTs and accuracy (i.e. percentage of correct responses) were recorded to specifically investigate the SAE. Because in HLT and IDVT participants were requested to indirectly and directly judge bodily identity, HLT was always conducted before IDTV.

**Fig 1:**
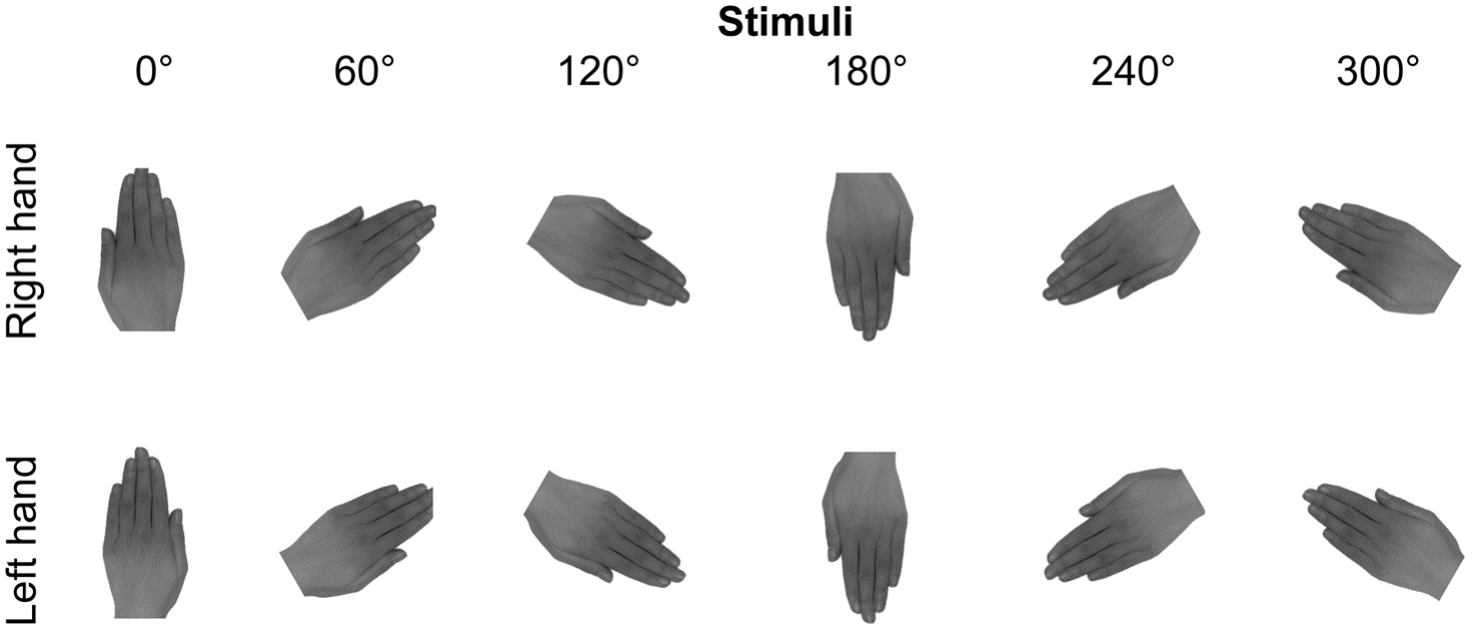
Stimuli. Examples of hand stimuli and orientations displayed in hand laterality and identity discrimination visual tasks, in case of both self- and other-stimuli.

Prior to the HLT, palm-down left and right hands of each participant were photographed using a high-resolution digital camera (Nikon Coolpix L830). Pictures were taken in a controlled environment (constant artificial light), where the camera was arranged in a permanent position, and digitally manipulated using GIMP (www.gimp.org). They were cropped, placed on a white background, and desaturated to get grey-scale images. Finally, hand pictures were rotated in six different clockwise orientations (0°, 60°, 120°, 180°, 240°, 300°; see Fig 1). Images of hands were presented one at a time at the center of a computer screen in both the HLT and the IDVT. Participants comfortably sat in front of the screen (at a distance of 40 cm) with relaxed hands located on the keyboard. Trials were run on E-Prime 2.0 (Psychology Software Tools Inc.) and started with a fixation cross lasting 500 msec, followed by the stimulus. Trials ended when participants responded to the stimulus (see hand laterality judgment and bodily identity discrimination measures; maximal duration 4 sec) and showed left and right hands belonging to each participant in half of the trials (self-trials) and to someone else in the remaining trails (other-trials). Self- and other-stimuli were matched by physical similarity (due to age, amount of thinness, dimension, shape, and color). Other-trials illustrated hands of three different individuals based on Ferri’s paradigm, which ruled out the possibility that advantage in processing self-stimuli could be due to a more frequent presentation of participants’ own hands than others’ hands. A total of 288 trials were run in pseudo-random order. 72 trials for each of the following conditions were presented: self-right hands, self-left hands, other-right hands, other-left hands. Each orientation was randomly presented 12 times per condition.

### Measures

#### Socio-demographic characteristics and handedness

Socio-demographic characteristics included age, BMI and education. Handedness (i.e. hand preference) was measured through the Edinburgh Handedness Inventory [41], a self-report scale which assesses the person's right or left hand preference in everyday activities.

#### Eating disorder psychopathology

The Eating Disorder Inventory (EDI) -3 [43] is a clinical self-report scale which provides a standardized evaluation of symptomatology associated with EDs. All participants completed the Italian version of EDI-3, and the general Eating Disorder Risk (EDRC) subscale of the EDI-3 to obtain information on eating behaviors.

#### Body representation

No standard scales specifically assessing body schema in EDs are available in Italian, therefore assessment of body representation disturbances relied on self-report standard scales evaluating perception- and affective-related bodily experience. The BMI-based Silhouettes Test (BMI-ST; [44]) was administered to measure distortion in visual perception of one’s own body (as a result of the difference between the perceived BMI and the actual BMI). The Italian version of the Body Attitude Test (BAT; [45]) was filled out to measure attitude toward one’s body, and total scores on the test (BAT-TOT) were computed and used as indicator of affective alterations of body image (i.e. body dissatisfaction).

#### Hand laterality judgment

A hand laterality judgment was obtained from the HLT, whereby participants mentally rotated their own and someone else’s hands in order to accomplish the task.

Participants were instructed to evaluate the laterality of the observed hand by pressing a left response key with the left index finger, when the observed hand was judged to be ‘left’, and a right response key with the right index finger when the observed hand was judged to be ‘right’. As in prior studies [24, 25, 32, 46], response keys were not counterbalanced across participants.

#### Bodily identity discrimination

Bodily identity discrimination was assessed with the IDVT, to control whether putative group differences in the SAE were specifically related to motor simulation and body schema, as argued by Ferri and coworkers [32]. To do so, participants were asked to evaluate whether or not the displayed hand corresponded to their own hand. This required a visual analysis of the stimulus, without a motor mental rotation. The same stimuli and conditions as in the HLT were used, and participants were instructed to respond by pressing a previously assigned left or a right response key with their left and right index fingers, respectively. Response keys were counterbalanced across participants.

### Statistical analysis

Data on age, BMI, education, and measures of EDs psychopathology and body representation were compared between EDs and HC participants by means of t-tests for independent samples. Handedness was compared between groups by means of a chi-squared test.

Analyses on RTs and response accuracy from HLT and IDVT were carried out separately. A mixed Analysis Of Variance (ANOVA) was performed with the within subjects factors ‘stimulus identity’ (other vs. self), ‘stimulus laterality’ (preferred vs. not preferred), and ‘stimulus orientation’ (0° vs. 60° vs. 120° vs. 180° vs. 240° vs. 300°), and the between-subjects factor ‘group’ (HC vs. EDs). In the case of ‘stimulus laterality’, we considered participants’ laterality preference (i.e. handedness) − and not the actual stimulus laterality − as both right- and left-handed participants were recruited. Effect sizes (Ƞ^2^_p_) are reported, and in case of significant effects pairwise post-hoc comparisons were performed using the Newman—Keuls procedure [47, 48].

In the case of the HLT, exploratory analyses were carried out on the SAE given the preliminary nature of our study. We computed the SAE size, i.e. the temporal difference in processing other- vs. self-stimuli (RTs_[other-self]_). We then carried out correlation analyses between SAE size and BMI-ST and BAT-TOT scores in EDs, in order to explore any relationship between body schema and image, as body representation was only measured by means of body image-related scales.

Statistical analyses were carried out using STATISTICA (StatSoft, www.statsoft.com) and SPSS (IBM, www.ibm.com). Significance level was set at p≤0.05.

## Results

### Socio-demographic characteristics and handedness

Participants with EDs and HCs did not differ in age, BMI, education, and handedness (see Table 1).

### Eating disorder- and body representation-related measures

Participants with EDs had higher scores on the EDRC, BMI-SMT, and BAT-TOT compared to controls, as expected (Table 1).

### Hand laterality judgement

#### RTs

**Fig 2:**
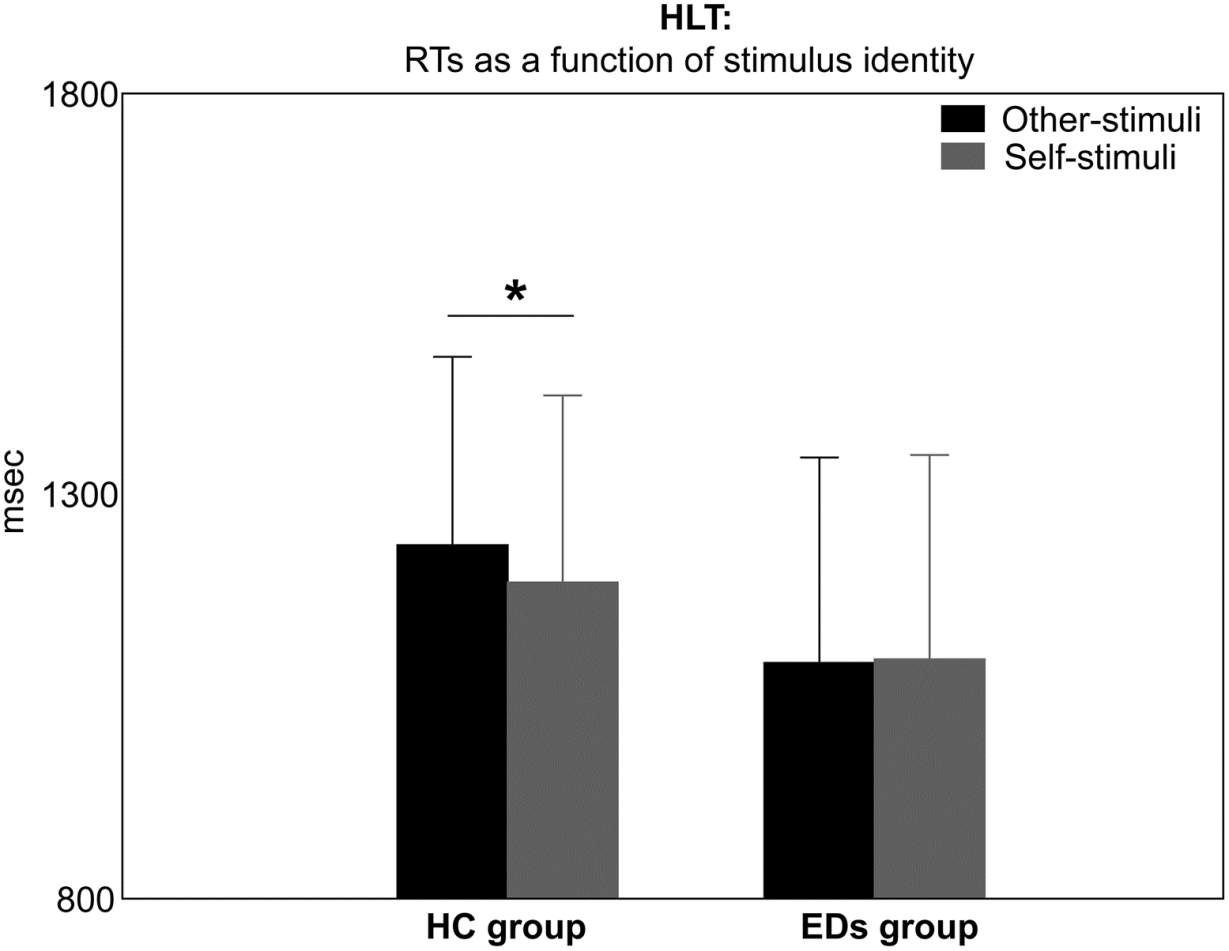
Self-Advantage Effect (SAE) in the Hand Laterality Task (HLT). Mean Response Times (RTs) as a function of stimulus identity in the HLT are reported for Healthy Control (HC) participants and participants with Eating Disorders (EDs). A temporal advantage in processing self-stimuli, compared to other-stimuli (SAE), only arose in the HC group, whereas patients with EDs processed self- and other-stimuli using the same amount of time. The horizontal line represents the significant post-hoc comparison. Bars are SE.

ANOVA on RTs revealed that the interaction between ‘group’ and ‘stimulus identity’ was significant (F(1,31) = 5.6, p = 0.02, Ƞ^2^_p_ = 0.1; see Table 2), and post-hoc tests (p<0.01) showed a decrease in RTs when processing self-stimuli (1192±232 msec), as opposite to other-stimuli (1239±233 msec), in the case of HCs only. In contrast, patients with EDs took the same amount of time when asked to mentally rotate other- (1092±256 msec) and self-stimuli (1097±254 msec; Fig 2 and Table 3).

**Table 2:** Response Times (RTs) and accuracy during the Hand Laterality Task (HLT) and the Identity Discrimination Visual Task (IDVT).

	HLT		IDVT	
Factors/Interactions	RTs (msec)	Accuracy (% of correct responces)	RTs (msec)	Accuracy (% of correct responces)
**Group**	F(1,31) = 1.5, p = 0.2; Ƞ^2^_p_ = 0.05	F(1,31) = 0.006, p = 0.9; Ƞ^2^_p_<0.001	F(1,31) = 0.8, p = 0.3; Ƞ^2^_p_ = 0.03	F(1,31) = 1.4, p = 0.2; Ƞ^2^_p_ = 0.04
**(Stimulus) identity**	F(1,31) = 3.8, p = 0.06; Ƞ^2^_p_ = 0.1	F(1,31) = 1.4, p = 0.2; Ƞ^2^_p_ = 0.04	F(1,31) = 0.003, p = 0.9; Ƞ^2^_p_<0.001	F(1,31) = 0.8, p = 0.3; Ƞ^2^_p_ = 0.03
**(Stimulus) orientation**	F(5,155) = 116.9, p<0.001*;**Ƞ**^**2**^_**p**_ **=** **0.8**	F(5,155) = 16.6, p<0.001*;** Ƞ**^**2**^_**p**_ **=** **0.3**	F(5,155) = 1.4, p = 0.2; Ƞ^2^_p_ = 0.04	F(5,155) = 3.8, p<0.01*;**Ƞ**^**2**^_**p**_ **=** **0.1**
**(Stimulus) laterality**	F(1, 31) = 24.7, p<0.001*;**Ƞ**^**2**^_**p**_ = **0.4**	F(1,31) = 0.002, p = 0.9; Ƞ^2^_p_<0.001	F(1,31) = 0.7, p = 0.4; Ƞ^2^_p_ = 0.02	F(1,31) = 2, p = 0.2; Ƞ^2^_p_ = 0.06
Identity* Group	F(1,31) = 5.6, p = 0.02*;**Ƞ**^**2**^_**p**_ **=** **0.1**	F(1,31) = 1.7, p = 0.2; Ƞ^2^_p_ = 0.05	F(1,31) = 0.2, p = 0.6; Ƞ^2^_p_ = 0.008	F(1,31) = 0.1, p = 0.7;Ƞ^2^_p_ = 0.003
Laterality* Group	F(1,31) = 0.6, p = 0.4; Ƞ^2^_p_ = 0.02	F(1,31) = 1.5, p = 0.2; Ƞ^2^_p_ = 0.05	F(1,31) = 0.3, p = 0.6; Ƞ^2^_p_ = 0.009	F(1,31) = 6.5, p = 0.01*;**Ƞ**^**2**^_**p**_ **=** **0.2**
Orientation* Group	F(5,155) = 1.5, p = 0.2; Ƞ^2^_p_ = 0.05	F(5,155) = 1.6, p = 0.1; Ƞ^2^_p_ = 0.05	F(5,155) = 0.3, p = 0.9; Ƞ^2^_p_ = 0.04	F(5,155) = 2.1, p = 0.07; Ƞ^2^_p_ = 0.06
Identity* Laterality	F(1,31) = 0.1, p = 0.7; Ƞ^2^_p_ = 0.004	F(1,31) = 0.8, p = 0.3; Ƞ^2^_p_ = 0.02	F(1,31) = 0.02, p = 0.9; Ƞ^2^_p_ = 0.001	F(1,31) = 0.6, p = 0.4; Ƞ^2^_p_ = 0.02
Identity* Orientation	F(5,155) = 1, p = 0.4; Ƞ^2^_p_ = 0.03	F(5,155) = 0.3, p = 0.8; Ƞ^2^_p_ = 0.01	F(5,155) = 2.9, p = 0.03*;**Ƞ**^**2**^_**p**_ **=** **0.1**	F(5,155) = 0.9, p = 0.4; Ƞ^2^_p_ = 0.03
Laterality* Orientation	F(5,155) = 3.4, p = 0.02*;**Ƞ**^**2**^_**p**_ **=** **0.1**	F(5,155) = 5.7, p<0.01*;**Ƞ**^**2**^_**p**_ **=** **0.2**	F(5,155) = 0.2, p = 0.07; Ƞ^2^_p_ = 0.07	F(5,155) = 1.3, p = 0.2; Ƞ^2^_p_ = 0.04
Identity* Laterality* Group	F(1,31) = 1.7, p = 0.2; Ƞ^2^_p_ = 0.05	F(1,31) = 0.1, p = 0.7; Ƞ^2^_p_ = 0.004	F(1,31) = 0.8, p = 0.4; Ƞ^2^_p_ = 0.001	F(1,31) = 0.8, p = 0.4; Ƞ^2^_p_ = 0.02
Identity* Orientation* Group	F(5,155) = 1, p = 0.3; Ƞ^2^_p_ = 0.03	F(5,155) = 0.8, p = 0.4; Ƞ^2^_p_ = 0.03	F(5,155) = 0.3, p = 0.8; Ƞ^2^_p_ = 0.008	F(5,155) = 0.5, p = 0.6; Ƞ^2^_p_ = 0.02
Laterality* Orientation* Group	F(5, 155) = 0.5, p = 0.7; Ƞ^2^_p_ = 0.02	F(5,155) = 0.5, p = 0.7; Ƞ^2^_p_ = 0.01	F(5,155) = 1.2, p = 0.3; Ƞ^2^_p_ = 0.04	F(5,155) = 0.7, p = 0.6; Ƞ^2^_p_ = 0.02
Identity* Laterality* Orientation* Group	F(5,155) = 0.2, p = 0.9; Ƞ^2^_p_ = 0.005	F(5,155) = 0.7, p = 0.6; Ƞ^2^_p_ = 0.02	F(5,155) = 0.3, p = 0.8; Ƞ^2^_p_ = 0.01	F(5,155) = 0.4, p = 0.8; Ƞ^2^_p_ = 0.01

A summary of the results of the ANOVAs is presented. Bold values index significant effects.

*Mean values for significant effects are reported in Table 3.

**Table 3:** Mean values of Response Times (RTs) and accuracy for significant effects in the ANOVAs in the Hand Laterality Task (HLT) and the Identity Discrimination Visual Task (IDVT).

	HLT		IDVT	
Signicant Factors/Interactions	RTs (msec)	Accuracy (% of correct responces)	RTs (msec)	Accuracy (% of correct responces)
**(Stimulus) Laterality**	pref = 1123±168 not pref = 1187±178			
**(Stimulus) orientation**	0° = 950±98 60° = 980±91 120° = 1263±124 180° = 1555±120 240° = 1215±115 300° = 967±86	0° = 95±1 60° = 96±1 120° = 93±2 180° = 85±5 240° = 94±2 300° = 96±1		0° = 88±4 60° = 88±4 120° = 84±5 180° = 85±5 240° = 85±4 300° = 86±4
Identity* Group	HC-other = 1239±233 HC-self = 1192±232 EDs-other = 1092±256 EDs-self = 1097±254			
Laterality* Group				HC-pref = 88±9 HC-not pref = 89±11 EDs-pref = 86±10 EDs-not pref = 81±12
Identity* Orientation			other-0° = 1061±70 other-60° = 1090±63 other-120° = 1138±72 other-180° = 1128±75 other-240° = 1127±77 other-300° = 1034±65 self-0° = 1068±75 self-60° = 1091±75 self-120° = 1099±72 self-180° = 1074±68 self-240° = 1114±73 self-300° = 1150±82	
Laterality* Orientation	pref-0° = 916±69 pref-60° = 970±67 pref-120° = 1279±100 pref-180° = 1510±86 pref-240° = 1145±77 pref-300° = 919±56 not pref-0° = 984±75 not pref-60° = 990±68 not pref-120° = 1248±82 not pref-180° = 1600±88 not pref-240° = 1285±93 not pref-300° = 1015±71	pref-0° = 97±1 pref-60° = 95±2 pref-120° = 91±2 pref-180° = 82±4 pref-240° = 96±1 pref-300° = 98±.6 not pref-0° = 93±2 not pref-60° = 98±2 not pref-120° = 95±2 not pref-180° = 88±4 not pref-240° = 92±3 not pref-300° = 95±2		

Mean values and SE for significant effects in the ANOVAs are reported.

**Fig 3:**
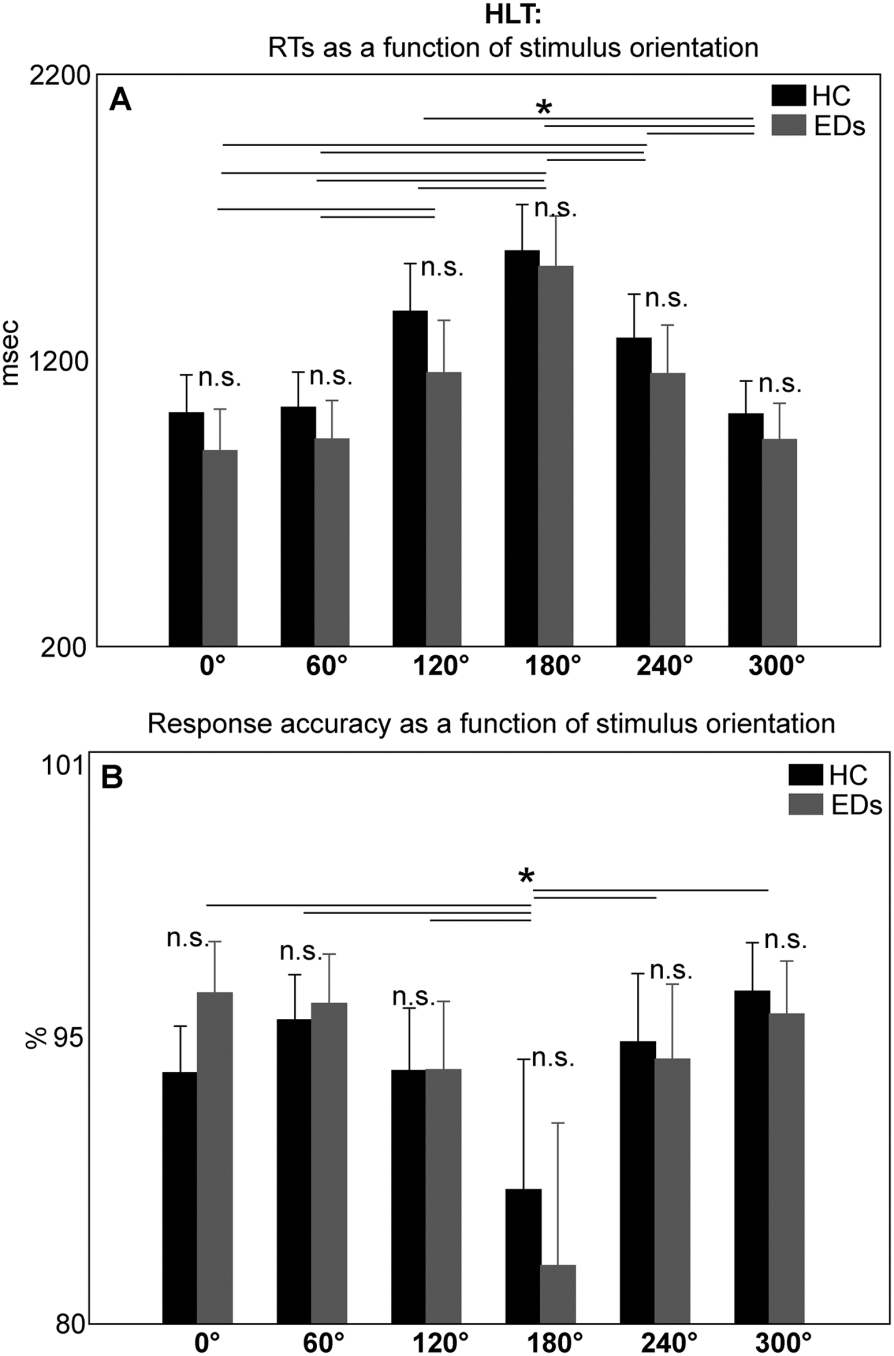
Response patterns as a function of stimulus orientation in the Hand Laterality Task (HLT). Overall Response Times (RTs; panel A) and accuracy (panel B) are significantly modulated by the amount of hand rotation, in both the EDs and the HC groups, in a manner compatible with the occurrence of motor mental rotation processes. Horizontal lines represent the significant post-hoc comparison. Bars are SE.

Interestingly, in the case of the HLT results of the ANOVA showed that RTs were significantly modulated by the amount of stimulus rotation (i.e. ‘stimulus orientation’ main effect, F(5,155) = 116.9, p<0.001, Ƞ^2^_p_ = 0.8; see Table 2) in both EDs and HC groups. More specifically, an increase in RTs up to 180° (1555±120 msec) was observed, followed by a decrease. Post-hoc comparisons (p<0.001) showed that all contrasts were significant, except for RTs when stimuli were oriented at 0° (950±98 msec) vs. 60° (980±91 msec), 0° vs. 300° (967±86 msec) and 300°, 60° vs. 300°; 120° (1263±124 msec) vs. 240° (1215±115 msec; Fig 3A and Table 3). In other words, RTs generally increased when the angular displacement between the observed and the real hand was greater. The main effect of ‘stimulus laterality’ was also significant (F(1, 31) = 24.7, p<0.001, Ƞ^2^_p_ = 0.4; see Tables 2 and 3) and a temporal gain when processing pictures showing participants’ preferred laterality (1123±168 msec), as opposed to not preferred laterality (1187±178 msec) was observed, in both EDs and HC groups. The interaction between ‘stimulus laterality’ and ‘stimulus orientation’ significantly modulated RTs pattern (F(5,155) = 3.4, p = 0.02, Ƞ^2^_p_ = 0.1; see Table 2). Post-hoc analysis (p<0.05) revealed, in both EDs and HC groups, a significant increase of RTs when processing not-preferred laterality-hands vs. preferred laterality-hand displayed at 180° (1600±88 vs.1510±86 msec), 240° (1285±93 vs. 1145±77 msec) and 300° (1015±71 vs. 919±56 msec; see Table 3). All results are reported in Table 2 and mean values for significant effects in Table 3.

#### Accuracy

In the case of the HLT, response accuracy was significantly affected by ‘stimulus orientation’ (F (5,155) = 16.6, p<0.001, Ƞ^2^_p_ = 0.3; see Table 2), and it was modulated by the amount of stimuli rotation in both EDs and HC groups. Post-hoc comparisons (p<0.001) showed that response accuracy significantly decreased for stimuli at 180° (85±5%; i.e. when the angular displacement between the observed and the real hand was extreme), as opposed to all the other orientations (0° = 95±1%; 60° = 96±1%; 120° = 93±2%; 240° = 94±2%; 300° = 96±1%; Fig 3B and Table 3).

The interaction between ‘stimulus laterality’ and ‘stimulus orientation’ was also significant in the case of response accuracy (F (5,155) = 5.7, p<0.01, Ƞ^2^_p_ = 0.2; see Table 2), and post-hoc analysis (p<0.05) showed a decrease in response accuracy when processing stimuli at 180° displayed at the preferred laterality (82±4%) as opposed to the not preferred laterality (88±4%). This occurred in both the EDs and HC groups (see Table 3).

No other group differences and effects significantly affected response accuracy. All results are reported in Table 2 and mean values for significant effects in Table 3.

No significant correlations emerged between SAE size and BMI-ST scores and between SAE size and BAT-TOT scores in EDs group (BMI-ST: r = -0.05, p = 0.8; BAT-TOT: r = 0.4, p = 0.1).

### Bodily identity discrimination

#### RTs

**Fig 4:**
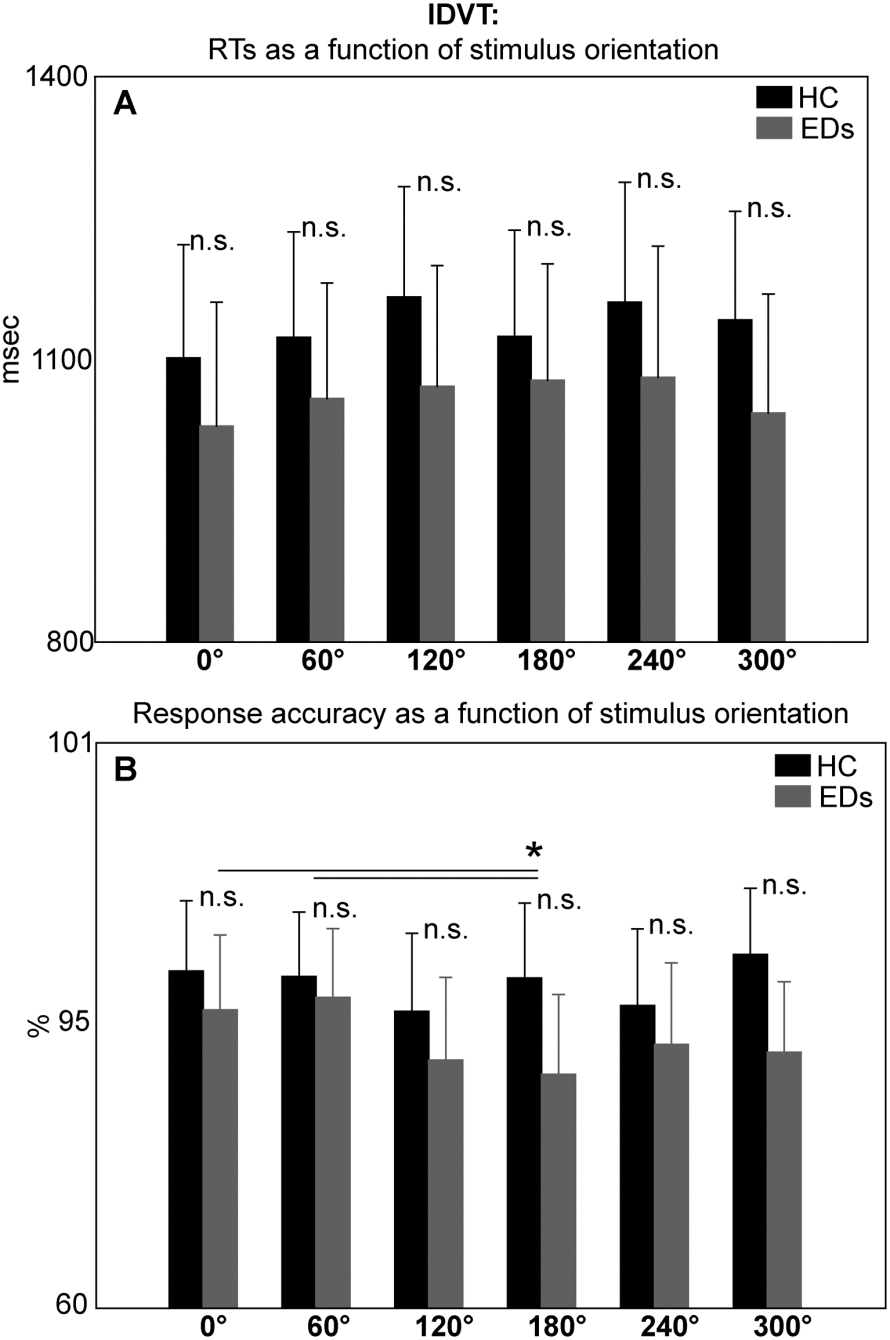
Response patterns as a function of stimulus orientation in the Identity Discrimination Visual Task (IDVT). Overall Response Times (RTs; panel A) and accuracy (panel B) are not modulated by the amount of hand rotation in a manner compatible with the activation of a motor mental rotation, neither in the EDs group nor in the HC groups. This pattern of responses suggests the actual activation of visual analysis processes. Horizontal lines represent the significant post-hoc comparison. Bars are SE.

In the case of the IDVT the ANOVA on RTs revealed no significant effects of either main factors or statistical interactions, except for the interaction between ‘stimulus identity’ and ‘stimulus orientation’ (F (5,155) = 2.9, p = 0.03, Ƞ^2^_p_ = 0.1; see Table 2). However, post-hoc analyses (p<0.05) only showed a significant increase in RTs when participants processed self-stimuli oriented at 300° (1150±82 msec) as opposed to other-stimuli at 300° (1034±65 msec). No other comparisons were significant. Therefore in the case of the IDVT RTs were not modulated by ‘stimuli orientation’ in a manner compatible with the occurrence of motor mental rotation processes as in the HLT (Fig 4A and Table 3).

No other group differences and effects significantly affected RTs. All results are reported in Table 2 and mean values for significant effects are reported in Table 3.

#### Accuracy

The ANOVA performed on response accuracy of the IDVT revealed that ‘stimulus orientation’ was significantly associated with accuracy (F (5,155) = 3.8, p<0.01, Ƞ^2^_p_ = 0.1; see Table 2). However, post-hoc comparisons (p<0.05) showed that accuracy only decreased in association with stimuli at 180° (85±5%) as opposite to those at 0° (88±4%) and 60° (88±4%; Fig 4B and Table 3).

The interaction between ‘group’ and ‘stimulus laterality’ was also significant (F (1,31) = 6.5, p = 0.01, Ƞ^2^_p_ = 0.2; see Tables 2 and 3). Post-hoc comparisons (p<0.05) showed that while HCs were equally accurate in visually discriminating between their own and someone else’s hands, regardless of whether the preferred (88±9%) or not preferred hand laterality (89±11%) was displayed, patients with EDs showed a selective worsening in accuracy when hands displayed at the not preferred laterality vs. the preferred laterality (81±12% vs. 86±10%) were processed.

No other group differences and effects significantly affected response accuracy. All results are reported in Table 2 and mean values for significant effects are reported in Table 3.

## Discussion and conclusion

This study focused on preliminarily investigating the high-level motor functions involved in body schema and in bodily self-representation in EDs, relevant to the most recent updates in the field of motor cognition [15, 31–34]. The ability to implicitly recognize and take advantage of one’s own body representation (i.e. SAE, [36, 37]) was specifically explored using the HLT [24, 25, 36, 37], a task in which patients with EDs and HCs observed their own and someone else’s hands in a series of rotations, and were required to mentally rotate the displayed hands in order to provide a laterality judgment. When a motor mental rotation occurs, RTs and response accuracy respectively increase and decrease as a function of the increase in the amount of angular displacement between the observer’s real hand and the presented hand [24, 25, 36, 37]. The HLT involves motor simulation [24, 25, 40, 46] and body schema manipulation [20–25]. In the case of the HLT of the present study, a motor mental rotation occurred in both patients with EDs and HCs, whose RTs and accuracy varied similarly and as a function of stimuli orientation. More specifically, an increase/decrease in the RTs/response accuracy was observed when the amount of displacement between participants’ and presented hands was greater. Moreover, in the HLT patients with EDs and HCs generally handled stimulus laterality in a similar manner, and their RTs and accuracy patterns varied in a comparable manner when the preferred hand was processed, showing thus analogous basic motor skills. However, the main finding of the present study was that, although patients with EDs were not impaired in basic motor manipulation of body schema to perform a mental rotation they selectively differed from controls in their ability to process self-stimuli vs. non-self-stimuli. In other words, patients with EDs did not show a SAE, as they took the same amount of time when mentally rotating their own and someone else’s hand. This preliminarily suggests that patients with EDs do not implicitly recognize their own body, as individuals who have a spared body representation do [32]. At a glance, a possible explanation for this finding could be that patients with EDs are generally impaired in the ability to discriminate between their own and someone else’s body. However, results of the IDVT, in which participants were requested to directly and visually determine whether the shown hand belonged to them or someone else do not confirm this hypothesis. In the case of the IDVT statistical analyses showed that RTs and accuracy patterns did not vary in a manner compatible with the actual performing of a motor mental rotation, and no differences arose when processing self- and other-stimuli across groups. Therefore, an inability to process bodily identity in relation to motor simulation might explain our findings, in line with previous hypotheses [31, 32]. This view is in line with Urgesi and colleagues’ proposal that a maladaptive body schema -related bodily self-representation exists in EDs [30].

According to some recent hypotheses in the field of motor cognition, body schema is totally motor in nature [15, 31, 32], and sense of ownership, i.e. ‘the perceptual status of one's own body’–which is strongly related to the ability to distinguish the bodily self from other’s bodies − directly depends on the motor system [31, 49]. Along these lines, the selective impairment in SAE we observed in patients with EDs might reflect a specific weakening in motor functions responsible for the sense of body, but not for basic kinematic topographies shared by mental rotation and actual action execution [50–53]. Obviously this last dichotomy should be further investigated using specifically designed tasks, but it is worth noting that empirical investigations on the exact role of the motor system in body representation disturbances in EDs are still lacking. Relevant to this are data by Candini and colleagues [54], who used our same paradigm and, similarly to us, found that a sample of right-brain damaged patients showed a selective deficit in implicitly processing self-body stimuli when their lesions mainly encompassed subcortical structures implicated in motor functions. This evidence strengthens the idea that the motor system is in fact involved in body representation disturbances in EDs, and encourages further investigations using neuroimaging techniques.

Generally speaking, the present study is the first to investigate higher-level functions of the motor system in EDs, hence extension and replication are required. However, our findings add to the evidence that patients with EDs show an atypical body representation when performing other paradigms [55], which quantitatively measure the sense of one’s own body [56]. Much research has in fact showed that multisensory integration leading to the experience of the body as one’s ‘own’ is linked to the possibility of performing actions with a specific body part [57–59]. Feeling of ownership and body awareness are associated with the activation of the premotor cortex [60–62].

Our findings differ from those of Urgesi and colleagues [30], as we found a selective—and not an overall—difficulty in manipulating body schema in EDs. However, although both involved a laterality judgement, tasks used in the two studies are not fully comparable, as they entailed different stimuli, contrasts, and mental rotation transformations. Indeed, while in [30] participants were asked to perform a mental transformation across two possible orientations, and in relation to whole-body stylized figures, our task was almost entirely based on a motor mental rotation which principally elicited motor imagery processes related to hand postures (i.e. motor simulation). These lower-level body schema motor functions were in turn used to elicit the occurrence of SAE (i.e., bodily identity self-recognition), not taking the stimulus physical perspective. Alternatively, another possible explanation of the observed discrepancy might be the nature of EDs investigated in the two studies: Urgesi et al. [30] focused on BN, whereas our sample included mostly patients with AN. This might suggest that, although present in both AN and BN, deficits in body schema might be qualitatively different across EDs. Unfortunately, the number of patients with BN in our study was too low to perform further investigations to make noteworthy inferences. However, differences between AN and BN should be examined more in depth, as neural circuits associated to body image disturbances might differ across diagnosis [63–65]).

In our study, SAE did not correlate with neither perceptual nor affective difficulties in body representation (i.e. body image). This is however in contrast with previous findings [2–4, 28, 66] suggesting that body image and body schema might not be totally independent. However, given the preliminary nature of the present investigation, this discrepancy may be due to the small sample size tested, which limits our power to detect a correlation. Further, larger investigations are therefore needed in this direction as well, as the relationship between body image and body schema has only minimally been explored in EDs, despite its importance in order to understand the neuro-functional underpinnings of body representation difficulties in EDs.

Patients with EDs showed better stimuli identity discrimination when processing the hand laterality compatible with their actual handedness. Because this only occurred in the IDVT and at the response accuracy level, we believe that this effect might be due to a tendency to visually analyze body stimuli in a more detail-based way, as previously shown [67, 68] in patients with AN. We propose that a similar mechanism might be at play in our study, whereby patients with EDs used a more constrained visual analysis, and better analyzed only one of the provided lateralities. This interpretation is in line with evidence that patients with AN have poorer set-shifting [69, 70].

The most important strength of the present study is that it investigated the role of the motor system in body schema impairment in EDs, and whether high-level motor functions may be compromised as part of body schema disturbances, for the first time following new insights in the field of motor cognition [15, 31–34]. We used body stimuli not related to weight and shape (i.e. hands; [56, 71]), and this is crucial, as it is unlikely that our paradigm might have triggered avoidance potentially affecting behavioral performance (see [72]). On the other hand, an important limitation of the present study is the relatively small sample size, which does not let us to make conclusive inferences. This is true in particular with regard to our choice to collapse together data on patients with AN and BN. Indeed, although AN and BN share difficulties in body representation [6] and body schema [26–30] their disturbances might be qualitatively different.

In conclusion, our study provides initial evidence that impairment in body schema characterizing EDs may be grounded in higher-level motor functions. Despite the preliminary nature of this study, our findings pave the way to a more detailed understanding of the nature of body representation disturbances in EDs, expanding it to the relatively neglected study of motor cognition.
